# Reverse transcription-quantitative polymerase chain reaction: description of a RIN-based algorithm for accurate data normalization

**DOI:** 10.1186/1471-2199-10-31

**Published:** 2009-04-15

**Authors:** Alexandre Ho-Pun-Cheung, Caroline Bascoul-Mollevi, Eric Assenat, Florence Boissière-Michot, Frédéric Bibeau, Dominic Cellier, Marc Ychou, Evelyne Lopez-Crapez

**Affiliations:** 1 INSERM U896, Val d'Aurelle Cancer Institute, Montpellier, France; 2 Merck Serono, Lyon, France; 3 Department of Oncobiology, Val d'Aurelle Cancer Institute, Montpellier, France; 4 Department of Biostatistics, Val d'Aurelle Cancer Institute, Montpellier, France; 5 Department of Medical and Digestive Oncology, Val d'Aurelle Cancer Institute, Montpellier, France; 6 Department of Pathology, Val d'Aurelle Cancer Institute, Montpellier, France

## Abstract

**Background:**

Reverse transcription-quantitative polymerase chain reaction (RT-qPCR) is the gold standard technique for mRNA quantification, but appropriate normalization is required to obtain reliable data. Normalization to accurately quantitated RNA has been proposed as the most reliable method for in vivo biopsies. However, this approach does not correct differences in RNA integrity.

**Results:**

In this study, we evaluated the effect of RNA degradation on the quantification of the relative expression of nine genes (*18S*, *ACTB*, *ATUB*, *B2M*, *GAPDH*, *HPRT*, *POLR2L*, *PSMB6* and *RPLP0*) that cover a wide expression spectrum. Our results show that RNA degradation could introduce up to 100% error in gene expression measurements when RT-qPCR data were normalized to total RNA. To achieve greater resolution of small differences in transcript levels in degraded samples, we improved this normalization method by developing a corrective algorithm that compensates for the loss of RNA integrity. This approach allowed us to achieve higher accuracy, since the average error for quantitative measurements was reduced to 8%. Finally, we applied our normalization strategy to the quantification of *EGFR*, *HER2 *and *HER3 *in 104 rectal cancer biopsies. Taken together, our data show that normalization of gene expression measurements by taking into account also RNA degradation allows much more reliable sample comparison.

**Conclusion:**

We developed a new normalization method of RT-qPCR data that compensates for loss of RNA integrity and therefore allows accurate gene expression quantification in human biopsies.

## Background

Reverse transcription-quantitative polymerase chain reaction (RT-qPCR) is the most sensitive method for mRNA quantification [1-4] as it allows the detection of rare transcripts and the observation of small variations in gene expression. Quantification of mRNA by RT-qPCR can be either absolute or relative. Absolute quantification gives the precise copy number of a target mRNA, but requires the construction of a calibration curve using standards of known concentration. On the other hand, relative quantification expresses the target quantity for an experimental sample as an n-fold difference relative to a calibrator. This is the method of choice to compare changes in mRNA expression between different samples. However, it requires data normalization in order to obtain biologically relevant results [5]. Generally this involves the use of one or several housekeeping genes, whose expression is assumed to be stable between individuals, experimental conditions or physiological states.

In molecular oncology, pre-therapeutic biopsies are interesting material for gene expression studies that aim at identifying prognostic or predictive molecular markers. However, it has been suggested that housekeeping genes should not be used for normalization when studies involve biopsies, since they exhibit large expression variability between individuals [6]. As an alternative, normalization to accurately quantitated total RNA has been proposed [4] and then validated in breast cancer biopsies [6]. This method relies on the precise measurement of the template RNA concentration [6,7] in order to ensure that equal amounts of RNA are used for reverse transcription (RT). Nevertheless, this may not be sufficient to allow reliable comparison among samples. Indeed, variations in the template RNA quality can introduce significant differences in subsequent RT-qPCR results [8]. RNA quality encompasses both its purity (absence of inhibitors) and its integrity (absence of degradation). Variability is mostly related to RNA integrity, as its degradation may greatly affect the measured gene expression levels [8,9]. Besides, previous studies suggested that there is a linear relation between gene expression measurement and RNA degradation [10-12]. However, to date, RNA integrity has not been taken into account for normalization of gene expression to total RNA.

The aim of this work was to evaluate the limits of normalization to accurately quantitated total RNA when using degraded samples and to improve this method by introducing a normalization factor that compensates for the loss of RNA integrity. For this purpose, using cell lines we first assessed the influence of RNA degradation on the quantification of the relative expression of nine genes (*18S*, *ACTB*, *ATUB*, *B2M*, *GAPDH*, *HPRT*, *POLR2L*, *PSMB6 *and *RPLP0*) that cover a wide expression spectrum. Our results show that RNA degradation could introduce large errors in gene expression measurements when data were normalized to total RNA. Therefore, to avoid unspecific variations due to RNA degradation, we developed a corrective algorithm that take into account the RNA integrity of each sample and we validated the proposed model through the quantification of *EGFR*, *HER2 *and *HER3 *mRNA in colon and breast cancer cell lines. Finally, we applied this strategy for the quantification of *EGFR*, *HER2 *and *HER3 *in rectal cancer biopsies.

## Results

### Quality Control of the RT-qPCR assay

We accurately measured the RNA concentration of the cell line samples using a tray cell system combined to a SAFAS UV mc2 spectrophotometer, and we verified sample purity by determining the A_260_/A_280 _ratio, which was always comprised between 2.0 and 2.1.

To assess sample-to-sample variations in the efficiency of both RT and PCR steps, we added a definite amount of an exogenous plant mRNA control (*CAB *mRNA, Table 1) to the RT reaction mix. After cDNA synthesis and amplification by qPCR, *CAB *expression was detected within a 1.5-fold range of concentration in all cell line samples. This suggests that there was no significant difference in the efficiency of both RT and PCR steps between samples.

**Table 1:** Genes examined

**Gene symbol**	**Gene name**	**GenBank accession no.**	**Primer sequences (5'→ 3')**	**Amplicon size (bp)**	**qPCR efficiency (%)**
***Control gene (assessment of RT-qPCR inhibitors)***					
*CAB*	*A. thaliana *chlorophyll a/b-binding protein	X56062	F: CCATTGCATTTGTTGAGCAC R: CAATTCCTCGAGCTTCTTGG	119	100
					
***Target genes – training set***					
*18S*	18S ribosomal RNA	X03205	F: GGCGCCCCCTCGATGCTCTTAG R: GCTCGGGCCTGCTTTGAACACTCT	89	98
*ACTB*	Beta-actin	NM_001101	F: CTGTGGCATCCACGAAACTA R: AGTACTTGCGCTCAGGAGGA	200	100
*ATUB*	Alpha tubulin	NM_006082	F: TTACCTCGACTCTTAGCTTGTCG R: GGATGGAGATGCACTCACG	107	92
*B2M*	Beta-2-microglobulin	NM_004048	F: CACCCCCACTGAAAAAGATG R: ATATTAAAAAGCAAGCAAGCAGAA	167	93
*GAPDH*	Glyceraldehyde-3-phosphate dehydrogenase	NM_002046	F: TGCACCACCAACTGCTTAGC R: GGCATGGACTGTGGTCATGAG	87	100
*HPRT*	Hypoxanthine phosphoribosyl transferase 1	NM_000194	F: TGATAGATCCATTCCTATGACTGTAGA R: AAGACATTCTTTCCAGTTAAAGTTGAG	126	94
*POLR2L*	Polymerase RNA II polypeptide L	NM_021128	F: CAACAAGTGGGAGGCTTACCT R: AGCTTCTCGATCAGGTCCAC	132	98
*PSMB6*	Proteasome subunit Y	NM_002798	F: GATACCGGGAAGACCTGATG R: AATGGCAAAGGACTGCCTTA	116	99
*RPLP0*	Ribosomal protein, large, P0	NM_001002	F: CACTGAGATCAGGGACATGTTG R: CTTCACATGGGGCAATGG	113	100
					
***Target genes – validation set***					
*EGFR*	Epidermal growth factor receptor	NM_005228	F: CTGGATCCCAGAAGGTGAGA R: GCCATCACGTAGGCTTCATC	111	100
*HER2*	v-erb-b2 erythroblastic leukemia viral oncogene homolog 2	NM_004448	F: CTCCTCCTCGCCCTCTTG R: AGCATGTCCAGGTGGGTCT	107	90
*HER3*	v-erb-b2 erythroblastic leukemia viral oncogene homolog 3	NM_001982	F: GTGGACTCGAGCAACATTGA R: CCGTACTGTCCGGAAGACAT	147	97

Forward and reverse primer sequences are indicated by "F" and "R", respectively.

### Effect of RNA degradation on relative gene expression

**Figure 1:**
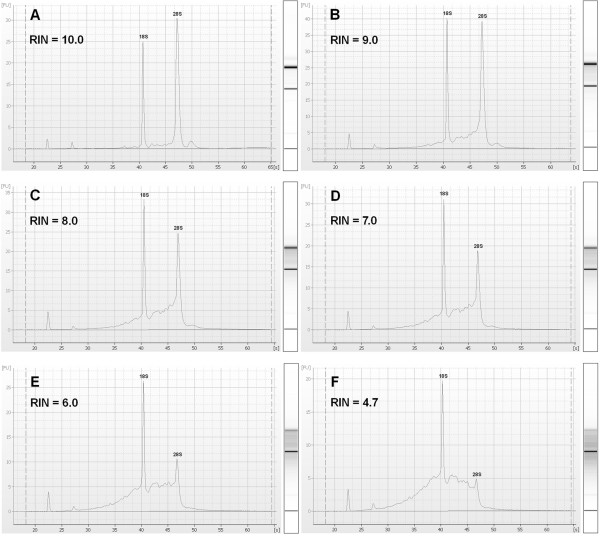
**Artificial degradation of HCT116 total RNA**. Several aliquots of a single HCT116 total RNA preparation were degraded at 70°C for different length of time and analyzed by the Agilent 2100 bioanalyzer. The resulting electrophoregrams and RIN after 0, 30, 51, 75, 140 and 165 minutes of incubation are shown in panel A, B, C, D, E and F, respectively.

**Figure 2:**
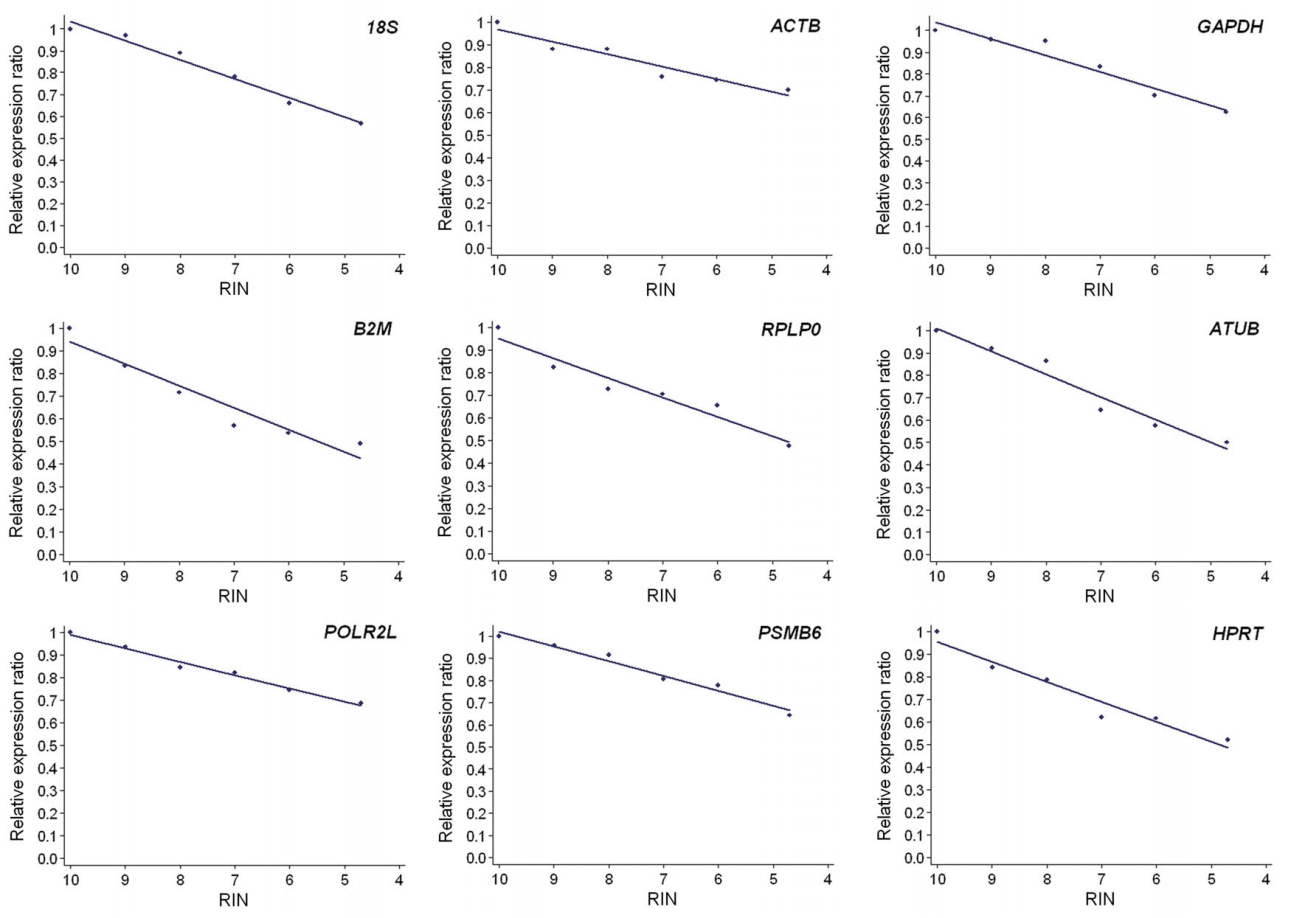
**Correlation between gene expression ratio and RIN in the HCT116 cell line**. The expression measurements of nine genes in increasingly degraded HCT116 samples are presented. The relationship between relative expression ratio and RIN was modeled by linear regression analysis.

To evaluate the limits of normalization to total RNA when using samples with impaired RNA integrity, we studied the effect of RNA degradation on gene relative expression. For that purpose, we aliquoted intact total RNA from HCT116, BxPC-3 and A427 cell lines and we gradually degraded each aliquot by hydrolysis at 70°C for different length of time ranging between 0 and 165 min. We monitored the degree of degradation with an Agilent 2100 bioanalyzer following the RNA integrity number (RIN) classification [13]. For each cell line, we obtained increasingly degraded samples, with RIN values going from 10 to 4.7. Figure 1 illustrates this artificial degradation by presenting the different degraded RNA samples obtained for the HCT116 cell line. Subsequently, using the artificially degraded RNA samples, we correlated the RIN of the input RNA with the relative transcription level of 9 target genes (Table 1: Target genes – training set), expressed as an n-fold difference relative to the intact (RIN = 10) samples. For all the studied genes and whatever the cell line considered, we found a linear relation between the RIN and the expression ratio (Figure 2 for the HCT116 cell line) (Table 2). The coefficients of determination (R^2^) ranged between 0.86 and 1.00. The mean slope value was 0.086 ± 0.025 (95% CI 0.076–0.096). The relatively low standard deviation observed indicates that all genes had comparable degradation profiles. Obviously, the lowest expression ratios were obtained for the most degraded samples. The minimum ratio observed was 0.48 for the RPLP0 gene in the HCT116 sample with RIN = 4.7 and it corresponded to a 2.08-fold difference between the intact sample ratio and the measured expression ratio. In other words, there was an error of 108% in the reported expression level of RPLP0 in this sample. Similarly, the maximum errors observed for samples with 5 ≤ RIN < 6, 6 ≤ RIN < 7, 7 ≤ RIN < 8 and RIN ≥ 8 reached 104%, 92%, 75% and 47%, respectively.

**Table 2:** Correlation between RIN and relative gene expression for 9 genes in the HCT116, BxPC-3 and A427 cell lines

**Gene**	**HCT116**			**BxPC-3**			**A427**		
	**Slope**	**Intercept**	**R^2^**	**Slope**	**Intercept**	**R^2^**	**Slope**	**Intercept**	**R^2^**
*18S*	0.09	0.16	0.98	0.14	-0.41	1.00	0.11	-0.16	0.94
*ACTB*	0.06	0.42	0.92	0.10	-0.03	0.94	0.06	0.41	0.99
*ATUB*	0.10	-0.01	0.96	0.14	-0.41	0.99	0.10	-0.01	0.95
*B2M*	0.10	-0.03	0.92	0.10	0.03	0.92	0.07	0.34	0.92
*GAPDH*	0.08	0.27	0.94	0.10	0.02	0.98	0.08	0.22	0.98
*HPRT*	0.09	0.08	0.94	0.10	0.05	0.86	0.08	0.16	0.96
*POLR2L*	0.06	0.4	0.98	0.06	0.43	0.92	0.04	0.60	0.97
*PSMB6*	0.07	0.35	0.97	0.09	0.14	0.99	0.05	0.53	0.99
*RPLP0*	0.09	0.09	0.94	0.11	-0.07	0.99	0.06	0.33	0.97

A regression analysis of the relationship between relative gene expression ratios and RIN was performed such that the expression ratio follows the relationship y = a × RIN + b, a being the slope and b the intercept. The coefficients of determination (*R*^2^) are also presented.

### Normalization of RNA degradation-related variations using a RIN-based algorithm

**Figure 3:**
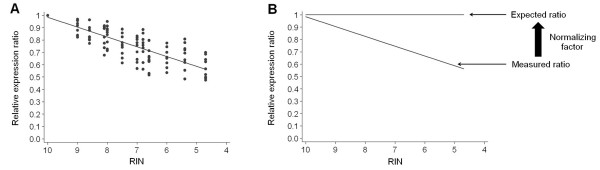
**Determination of a RIN-based normalizing factor**. (A) The measured expression ratios of 9 genes in HCT116, BxPC-3, and A427 samples with decreasing RNA integrity allowed the determination of an average gene degradation profile that follows the equation: relative expression ratio = 0.08 × RIN + 0.19. (B) A RIN-based normalizing factor was determined from the deviation between the average gene degradation and the line y = 1, which corresponds to the expected ratio for intact samples (RIN = 10).



To set up a normalizing factor that could compensate for the loss of RNA integrity, we first determined the average gene degradation profile based on the data we obtained for *18S*, *ACTB*, *ATUB*, *B2M*, *GAPDH*, *HPRT*, *POLR2L*, *PSMB6 *and *RPLP0 *in the increasingly degraded HCT116, BxPC-3 and A427 RNA samples (Figure 3). We then modeled our data by linear regression analysis of the mean measured ratios such that the average degradation profile followed the relationship y = a × RIN + b, where a = 0.08 and b = 0.19. Since each gene's transcription level was expressed as an n-fold difference relative to the RIN = 10 sample of the corresponding cell line, the expected ratio for intact samples (RIN = 10) corresponded to the line y = 1. The RIN-normalized ratio (R_RIN_) could then be calculated as follow:

To assess the validity of this normalization factor, we applied our model to the quantification of *EGFR*, *HER2* and *HER3* expression in LS174T (colon adenocarcinoma) and SKBr3 (breast carcinoma) samples displaying variable RNA integrity (Table 3). For each measure, the accuracy was greatly increased when using the corrective factor. While pre-normalized data exhibited errors up to 100% in gene quantification, the maximum error after normalization was below 25%. The mean error for the normalized ratios was 8.4% ± 6.6 (95% CI 5.6–11.2).

**Table 3:** Normalization of *EGFR*, *HER2 *and *HER3 *expression according to the RIN

**Cell line**	**RIN**	***EGFR***		***HER2***		***HER3***	
		**Measured ratio ** **(% error)**	**RIN-normalized ratio ** **(% error)**	**Measured ratio ** **(% error)**	**RIN-normalized ratio ** **(% error)**	**Measured ratio ** **(% error)**	**RIN-normalized ratio ** **(% error)**
LS174T	10.0	1.00 (0.0)	-	1.00 (0.0)	-	1.00 (0.0)	-
	8.0	0.75 (33.3)	0.91 (9.9)	0.76 (31.6)	0.93 (7.5)	0.83 (20.5)	1.01 (1.0)
	7.1	0.63 (58.7)	0.84 (19.0)	0.74 (35.1)	0.99 (1.0)	0.63 (58.7)	0.84 (19.0)
	6.6	0.60 (66.7)	0.85 (1.6)	0.63 (58.7)	0.89 (12.4)	0.62 (61.3)	0.87 (14.9)
	5.4	0.50 (100.0)	0.81 (23.5)	0.60 (66.7)	0.98 (2.0)	0.55 (81.8)	0.89 (12.4)
							
SKBr3	10.0	1.00 (0.0)	-	1.00 (0.0)	-	1.00 (0.0)	-
	7.9	0.84 (19.0)	1.03 (3.0)	0.80 (25.0)	0.99 (1.0)	0.85 (17.6)	1.05 (5.0)
	7.2	0.73 (37.0)	0.96 (4.2)	0.68 (47.0)	0.90 (11.1)	0.74 (35.1)	0.98 (2.0)
	5.9	0.64 (56.2)	0.98 (2.0)	0.61 (63.9)	0.93 (7.5)	0.72 (38.9)	1.10 (10.0)
	5.1	0.56 (78.6)	0.95 (5.2)	0.57(75.4)	0.96 (4.2)	0.63 (58.7)	1.07 (7.0)

*EGFR*, *HER2 *and *HER3 *gene transcription levels were assessed in LS174T and SKBr3 cell samples with decreasing RNA integrity and expressed as n-fold difference relative to the intact (RIN = 10) sample. RIN-normalized ratios were calculated according to the following formula: RIN-normalized ratio = Measured ratio + (Measured ratio × (1-(0.08 × RIN + 0.19))/(0.08 × RIN + 0.19)). The percent of error (% error) shows the accuracy of the estimated (i.e. measured or normalized) ratio and was calculated as follow: % error = ((expected ratio – estimated ratio)/estimated ratio) × 100%.

### Application of the RIN-based normalization factor in mRNA quantification of biopsy samples

**Figure 4:**
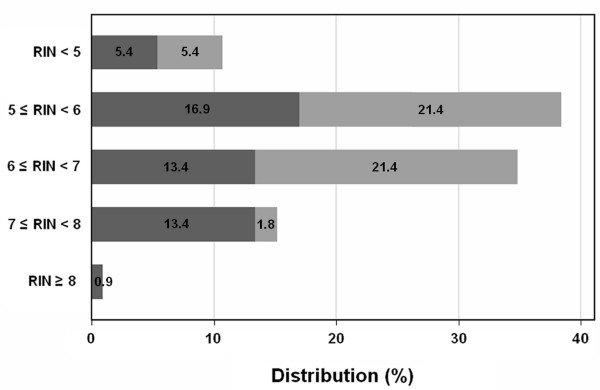
**RIN value frequency distribution for RNA isolated from 112 rectal biopsies**. Dark and light bars correspond to tumor and normal samples, respectively.

**Figure 5:**
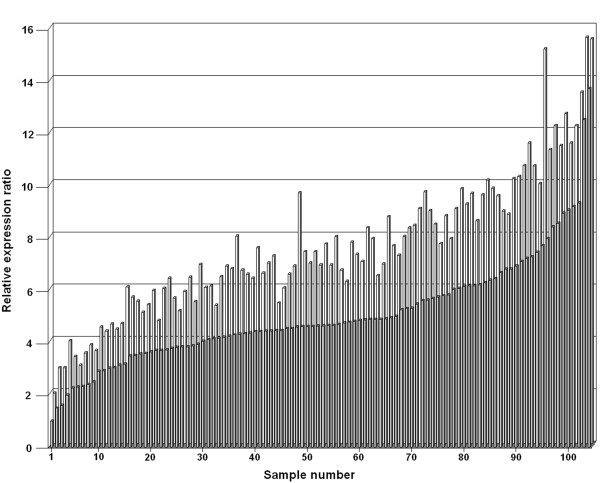
**Application of the RIN-based normalization factor for *EGFR *quantification in rectal biopsy samples**. The relative expression ratio corresponds to *EGFR *expression level, expressed as an n-fold difference relative to the sample with the lowest *EGFR *expression. Dark and light gray bars correspond to non-normalized and RIN-normalized relative expression ratios, respectively.

To evaluate the accuracy of normalization to total RNA of RT-qPCR data obtained from in vivo biopsies, we determined the RIN of 112 RNA samples isolated from 56 paired normal/tumor rectal tissues (Figure 4). The majority (73.2%) of samples were distributed in the 5 ≤ RIN < 6 and 6 ≤ RIN < 7 categories. One hundred and four RNA samples had sufficient RNA concentration for RT, and cDNA were obtained for these samples. Then, we compared the relative expression of *EGFR*, *HER2 *and *HER3 *in these samples (Figure 5 for *EGFR*) with and without the application of our RIN-based normalization factor. The mean fold-difference between non-normalized and RIN-normalized values was 1.52 ± 0.17 (95% CI 1.47–1.55) and ranged from 1.13 to 2.10. Moreover, our RIN-based algorithm allowed the exposure of some differences in gene expression levels among samples that could not have been seen otherwise. For instance, without normalization, sample 48 and 50 exhibited *EGFR *expression ratios of 4.59 and 4.61, respectively, which would lead to the conclusion that *EGFR* was expressed at similar levels in both samples. After normalization of variations due to RNA degradation using our corrective RIN-based algorithm, *EGFR *was more strongly expressed in sample 48 (ratio = 9.60) than in sample 50 (ratio = 6.91). Furthermore, variations in RNA integrity may generate misleading differences in gene expression measurements. Indeed, the non-normalized ratios of sample 95 (ratio = 7.71) and sample 100 (ratio = 9.07) suggested that the former exhibited a lower *EGFR *expression level, while the RIN-normalized ratios led to the opposite conclusion. *EGFR *expression was actually higher in sample 95 (ratio = 15.10) than in sample 100 (ratio = 11.51).

## Discussion

Normalization of gene expression levels to total RNA requires precise quantification of the RNA template. Several methods exist for measuring RNA concentrations, and we have previously discussed their respective advantages and drawbacks [14]. In this study, we determined total RNA concentration by measuring the optical density at 260 nm with a TrayCell system associated to a SAFAS UV mc2 spectrophotometer. This system offers sensitivity down to 2 ng/μl and allows the analysis of extremely small volumes (0.7–4 μl), which has the advantage of avoiding dilution errors. Once the sample concentration is accurately determined, the simplest way to normalize gene expression using total RNA is to ensure that equal amounts of input RNA are used for the RT reaction, all the more so that the cDNA yield is dependent on template abundance [5,15].

Normalization to total RNA also requires assessment of the presence of RT-qPCR inhibitors in samples [6,14]. These inhibitors, which may include reagents used during RNA isolation, or co-purified biological components [16,17], can reduce the efficiency of both RT and PCR and generate errors in the quantification results. In this study, we used an exogenous *CAB *mRNA control [18,19] that was co-reverse-transcribed with each sample RNA and then amplified by qPCR. Thus, any variation in *CAB *expression level would reflect variations in the efficiency of the RT and/or PCR steps. *CAB *showed a 1.5-fold variation range in our cell line cDNA samples, which is comparable to or even narrower than previously reported values for similar exogenous controls [6,19,20]. We conclude that in our samples and under our optimized RT-qPCR conditions, there was only a negligible effect of inhibitors on the RT and PCR efficiencies.

Bustin *et al. *[7] recommended normalization to accurately quantitated total RNA as the least unreliable method, and Tricarico *et al. *[6] validated it for breast biopsies [6]. However, little was known at that time about the accuracy of this approach when using degraded RNA samples. In this study, we assessed the effect of RNA degradation on the relative gene expression level measured by RT-qPCR in 3 different models, namely colorectal carcinoma (HCT116), pancreatic adenocarcinoma (BxPC-3) and lung adenocarcinoma (A427) cell lines. Different methods to degrade RNA have been described in the literature, including the use of RNase treatment [11], UV radiation [11], or thermal hydrolysis [21]. While these procedures are artificial and may differ from the natural degradation that occurs during sample handling, they allow producing a collection of RNA samples that are representative of all possible degrees of RNA degradation. Using thermal hydrolysis, we degraded total RNA isolated from HCT116, BxPC-3 and A427 cell lines. We thus obtained samples with decreasing integrity, with RIN values ranging from 10 (intact RNA) to 4.7 (highly degraded RNA), which corresponded to the range allowing reliable RT-qPCR quantification analysis [11]. Then, we measured the expression of *18S*, *ACTB*, *ATUB*, *B2M*, *GAPDH*, *HPRT*, *POLR2L*, *PSMB6 *and *RPLP0*, a group of genes that covers a wide expression range. Since all samples from a given cell line had the same transcriptome, the decrease in the measured gene expression ratios accurately reflected the effect of RNA degradation. Our data demonstrate that there is a linear correlation between the relative expression ratio of a gene and the RIN: the lower the RIN, the higher the decrease in the measured expression level. One should keep in mind that these results may be specific to the protocol used in this study. We have carefully designed our protocol in order to reduce the effects of RNA degradation and maximize the yield of the RT reaction. Specifically, we preferred random hexamers over oligo(dT) or specific primers, which are not appropriate for fragmented RNA [8], and we chose PCR product sizes smaller than 200 bp (Table 1), as short amplicons have been shown to be less dependent on RNA integrity [10]. Fleige *et al. *[11] have already tested the effect of artificial RNA degradation on gene expression for a limited number of genes (*18S*, *28S*, *ACTB *and *IL-1β*) in a large panel of human tissue-derived RNAs. Similarly to our results, they found a linear correlation between gene expression and RIN. However, in their study, this was not true for all tissue types. This may be imputed to differences between our experimental protocols. Specifically, they performed one-step RT-qPCR assays with specific primers, and chose longer PCR products (i.e., 198–338 pb). Tissues definitely show different sensitivity to RNA degradation, but for a givengene that is similarly expressed in two different tissues, the quantification of its expression using an optimized RT-qPCR protocol should be influenced only by the sample's degradation level (i.e. its RIN value), and not by the tissue type.

In our experiment, the most degraded samples exhibited up to 2-fold decrease in gene expression levels. This demonstrates that, for samples with RIN values down to 4.7, variations in RNA integrity may generate an error of approximately 100% in gene quantification. To address this issue, we asked whether it was possible to determine a RIN-based algorithm that normalizes the loss of RNA integrity in gene quantification. This implies the determination of the gene of interest's degradation profile. Since 1) it is hardly conceivable to model all possible degradation profiles in the short term and 2) the 9 training genes analyzed in this study showed similar degradation profiles, we chose to determine an average degradation profile based on the data we obtained for these genes in colon, pancreatic and lung cancer cells. Then, using this consensus profile, we calculated a normalizing factor that adjusted the RIN-dependent quantitative measure to the expected value for intact samples.

To assess the validity of this corrective algorithm, we applied the proposed normalization method to the quantification of *EGFR*, *HER2 *and *HER3 *in samples with decreasing RNA integrity obtained from two model-independent cancer cell lines (LS174T, colon; SKBr3, breast). Our results demonstrate that the developed approach greatly reduces RNA degradation-related variations for all genes in each sample. The use of the RIN-corrective algorithm lowered the maximum error in quantification from 100% to less than 25%, and an average error of less than 10% was obtained. Such accuracy is desirable, since minimal changes in gene expression levels can have important functional [22] or clinical [23] consequences.

For studies involving human biopsies, analysis of samples with variable RNA integrity is unavoidable as RNA is usually degraded during sample handling. Therefore, normalization of variations due to RNA degradation is of critical importance. In this study, we assessed the degradation level of 112 RNA extracted from 56 matching normal and tumor rectal biopsies pairs. Nearly 75% of samples showed RIN values comprised between 5 and 7 and our experiment with gradually degraded cell lines demonstrated that samples within this range of RIN could exhibit important errors in gene expression measurements. To assess the benefit of our RIN-based corrective algorithm, we measured the expression of *EGFR*, *HER2 *and *HER3 *in 104 of the 112 RNAs derived from biopsies and compared non-normalized and RIN-normalized ratios. Our data indicate that, without normalization, differences in sample RNA integrity could generate artificial up- or down-regulations that could lead to misleading interpretation of the results. Although our model will not fit perfectly each gene due to possible differences in degradation profiles, it will significantly reduce unspecific variations. Therefore, we recommend the use of our RIN-based corrective algorithm when normalizing gene expression measurements to accurately quantitated RNA. However, this requires the use of our RT protocol and the design of short PCR products (< 200 pb). To make this normalization process more user-friendly, we plan to develop a software program that normalizes target gene expression measurements according to the RIN value in an automatic manner.

## Conclusion

The precision and accuracy of gene expression measurements with RT-qPCR depend on the method used to normalize the data. In this study, we demonstrate that the use of total RNA for RT-qPCR normalization is limited when small differences in gene expression need to be detected. To achieve higher accuracy in RT-qPCR measurements, we improved this method by introducing a RIN-based corrective algorithm. This strategy should correct variations related to RNA degradation and allow accurate gene expression quantitation.

## Methods

### Patients' tissues and cell line

The human cancer cell lines HCT116, BxPC-3, A427, SKBr3 and LS174T were purchased from the American Type Culture Collection and cultured under standard conditions. Cells were harvested at 50% confluence, washed with phosphate buffered saline, and subsequently used for RNA extraction.

Fifty-six rectal cancer patients were included in this study between January 2006 and February 2008. For all patients, pre-therapeutic biopsies from paired normal/tumor rectal tissues were obtained by endoscopy. Biopsies were frozen at -80°C within 45 minutes and stored under this condition until extraction. The protocol was approved by the CPP of Saint-Eloi Hospital (Montpellier, France), a French Ethic committee for the protection of patients involved in biomedical research.

### RNA Isolation and Characterization

Total RNA was isolated using the RNeasy Mini Kit (Qiagen, Courtaboeuf, France) following the manufacturer's instructions. The extraction included a digestion step with DNase I to prevent subsequent amplification of genomic DNA. Total RNA concentration was determined by measuring the absorbance at 260 nm (A_260_) with the SAFAS UV mc2 spectrophotometer (Safas, Monaco, Monaco), using a TrayCell system (Hellma, Paris, France). Total RNA purity was verified by determining the A_260_/A_280 _ratio. RNA integrity was assessed by microcapillary electrophoresis with the RNA 6000 Nano LabChip kit (Agilent Biotechnologies, Massy, France) and the Agilent 2100 bioanalyzer (Agilent Biotechnologies), which assigns a RIN to each RNA electropherogram. This number ranges from 1 (completely degraded RNA sample) to 10 (intact RNA sample).

### Reverse transcription

For each sample, a 13-μl mix containing 1 μg total RNA, 150 ng of random hexamers (Promega, Charbonnieres, France), 1 μl of a 10 mM dNTP Mix (Invitrogen, Cergy Pontoise, France), and 0,3 pg of an exogenous plant mRNA spike (*A. thaliana *chlorophyll a/b-binding protein, CAB) (Stratagene, Amsterdam, The Netherlands) was heated at 65°C for 5 minutes. After cooling on ice, a 7 μl-reaction mix containing 1 μl of SuperScript™ III Reverse Transcriptase (200 U/μl) (Invitrogen), 4 μl of 5× First-Strand Buffer (Invitrogen), 1 μl of 0.1 M DTT (Invitrogen), and 1 μl of SUPERase. In™ (20 U/μl) (Ambion, Huntingdon, UK) was added. Then reverse transcription was performed in an Eppendorf^® ^Mastercycler^® ^(Eppendorf, Le Pecq, France) with an initial priming step at 25°C for 5 minutes, followed by cDNA synthesis at 50°C for 60 minutes. A final inactivation step at 70°C for 15 minutes completed the reaction.

### Quantitative real-time RT-PCR analysis

We developed quantitative SYBR green PCR assays for the 12 genes involved in this study and the spiked plant mRNA control (Table 1). Real-time PCR amplification was performed in a Rotor-Gene™ 6000 (Labgene, Archamps, France) using the ABsolute™ Blue QPCR SYBR^® ^Green Mix (ABgene, Courtaboeuf, France). PCR amplification were carried out in a 20-μl volume with the following cycling conditions: an enzyme activation step at 95°C for 15 minutes, followed by 40 cycles consisting of 15 seconds of denaturation at 95°C, 30 seconds of annealing at 58–64°C depending on primers, and 30 seconds of elongation at 72°C. The specificity of the amplified products was verified by melting curve analysis and agarose gel electrophoresis. For each qPCR run, a standard curve was generated using serial dilutions of a standard cDNA. Amplification efficiencies (E) were calculated from the slope of the standard curves according to the equation: E = 10 [-1/*slope*], and they ranged from 90% to 100%. To exclude between-run variations, all cDNA samples were tested in duplicate in the same analytical run along with a calibrator. A value of 1 was attributed to the calibrator and all gene expression levels were expressed as an n-fold difference relative to the calibrator, according to the relative standard curve method [24].

### Statistical analysis

All statistical analyses were performed with the STATA 10.0 software (StataCorp, College Station, TX).

## Abbreviations

qPCR: quantitative polymerase chain reaction; RIN: RNA integrity number; RT: reverse transcription.

## Authors' contributions

AH designed the study, performed the experiments, and wrote the manuscript. CB performed the statistical analyses and contributed to the content and writing of the paper. EA performed the endoscopic biopsies and provided the clinical samples. FBo and FBi collected and validated the biopsies. DC and MY managed and coordinated the project. EL supervised the design and execution of experiments and participated in the writing of the manuscript. All authors have read and approved the final manuscript.

## References

[B1] Wang T, Brown MJ (1999). mRNA quantification by real time TaqMan polymerase chain reaction: validation and comparison with RNase protection. Anal Biochem.

[B2] Orlando C, Pinzani P, Pazzagli M (1998). Developments in quantitative PCR. Clin Chem Lab Med.

[B3] Lockey C, Otto E, Long Z (1998). Real-time fluorescence detection of a single DNA molecule. Biotechniques.

[B4] Bustin SA (2000). Absolute quantification of mRNA using real-time reverse transcription polymerase chain reaction assays. J Mol Endocrinol.

[B5] Bustin SA, Benes V, Nolan T, Pfaffl MW (2005). Quantitative real-time RT-PCR – a perspective. J Mol Endocrinol.

[B6] Tricarico C, Pinzani P, Bianchi S, Paglierani M, Distante V, Pazzagli M, Bustin SA, Orlando C (2002). Quantitative real-time reverse transcription polymerase chain reaction: normalization to rRNA or single housekeeping genes is inappropriate for human tissue biopsies. Anal Biochem.

[B7] Bustin SA (2002). Quantification of mRNA using real-time reverse transcription PCR (RT-PCR): trends and problems. J Mol Endocrinol.

[B8] Bustin SA, Nolan T (2004). Pitfalls of quantitative real-time reverse-transcription polymerase chain reaction. J Biomol Tech.

[B9] Imbeaud S, Graudens E, Boulanger V, Barlet X, Zaborski P, Eveno E, Mueller O, Schroeder A, Auffray C (2005). Towards standardization of RNA quality assessment using user-independent classifiers of microcapillary electrophoresis traces. Nucleic Acids Res.

[B10] Fleige S, Pfaffl MW (2006). RNA integrity and the effect on the real-time qRT-PCR performance. Mol Aspects Med.

[B11] Fleige S, Walf V, Huch S, Prgomet C, Sehm J, Pfaffl MW (2006). Comparison of relative mRNA quantification models and the impact of RNA integrity in quantitative real-time RT-PCR. Biotechnol Lett.

[B12] Auer H, Lyianarachchi S, Newsom D, Klisovic MI, Marcucci G, Kornacker K (2003). Chipping away at the chip bias: RNA degradation in microarray analysis. Nat Genet.

[B13] Schroeder A, Mueller O, Stocker S, Salowsky R, Leiber M, Gassmann M, Lightfoot S, Menzel W, Granzow M, Ragg T (2006). The RIN: an RNA integrity number for assigning integrity values to RNA measurements. BMC Mol Biol.

[B14] Ho-Pun-Cheung A, Cellier D, Lopez-Crapez E (2008). [Considerations for normalisation of RT-qPCR in oncology.]. Ann Biol Clin (Paris).

[B15] Karrer EE, Lincoln JE, Hogenhout S, Bennett AB, Bostock RM, Martineau B, Lucas WJ, Gilchrist DG, Alexander D (1995). In situ isolation of mRNA from individual plant cells: creation of cell-specific cDNA libraries. Proc Natl Acad Sci USA.

[B16] Freeman WM, Walker SJ, Vrana KE (1999). Quantitative RT-PCR: pitfalls and potential. Biotechniques.

[B17] Nolan T, Hands RE, Ogunkolade W, Bustin SA (2006). SPUD: a quantitative PCR assay for the detection of inhibitors in nucleic acid preparations. Anal Biochem.

[B18] Steinau M, Rajeevan MS, Unger ER (2006). DNA and RNA References for qRT-PCR Assays in Exfoliated Cervical Cells. J Mol Diagn.

[B19] Steinau M, Rajeevan MS, Lee DR, Ruffin MT, Horowitz IR, Flowers LC, Tadros T, Birdsong G, Husain M, Kmak DC, Longton GM, Vernon SD, Unger ER (2007). Evaluation of RNA markers for early detection of cervical neoplasia in exfoliated cervical cells. Cancer Epidemiol Biomarkers Prev.

[B20] de Kok JB, Roelofs RW, Giesendorf BA, Pennings JL, Waas ET, Feuth T, Swinkels DW, Span PN (2005). Normalization of gene expression measurements in tumor tissues: comparison of 13 endogenous control genes. Lab Invest.

[B21] Mueller S (2008). Optimizing real-time quantitative PCR experiments with the Agilent 2100 bioanalyzer. Agilent Technologies – Application Note 5989-7730EN.

[B22] Doebley J, Lukens L (1998). Transcriptional regulators and the evolution of plant form. Plant Cell.

[B23] Yan H, Dobbie Z, Gruber SB, Markowitz S, Romans K, Giardiello FM, Kinzler KW, Vogelstein B (2002). Small changes in expression affect predisposition to tumorigenesis. Nat Genet.

[B24] ABI Relative quantitation of gene expression. User bulletin No 2 ABI prism 7700 Sequence Detection System PE Applied Biosystems.

